# Supplementary figures and images for: Investigating audience preferences within the hybrid competitive-comedic format of taskmaster UK
Source: PLoS One. 2025 Sep 10;20(9):e0331064. doi: 10.1371/journal.pone.0331064 (PMC12422456; doi:10.1371/journal.pone.0331064)

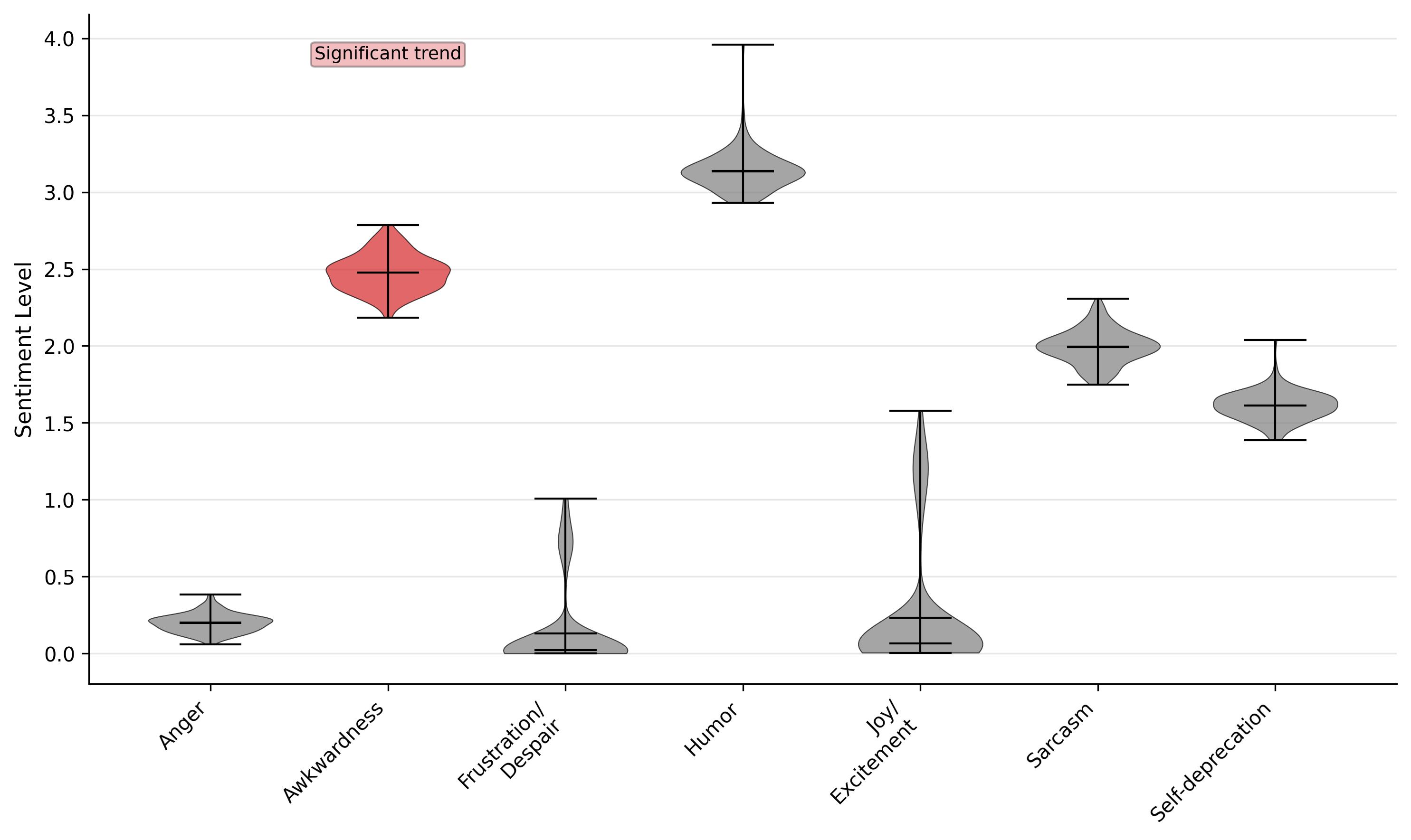

Supplement: S1 Fig — Box plots showing the distribution of each sentiment category across the full 154-episode corpus. While mean levels remain stable, awkwardness exhibits a broader distribution in later series. (PNG) [file pone.0331064.s001.png]

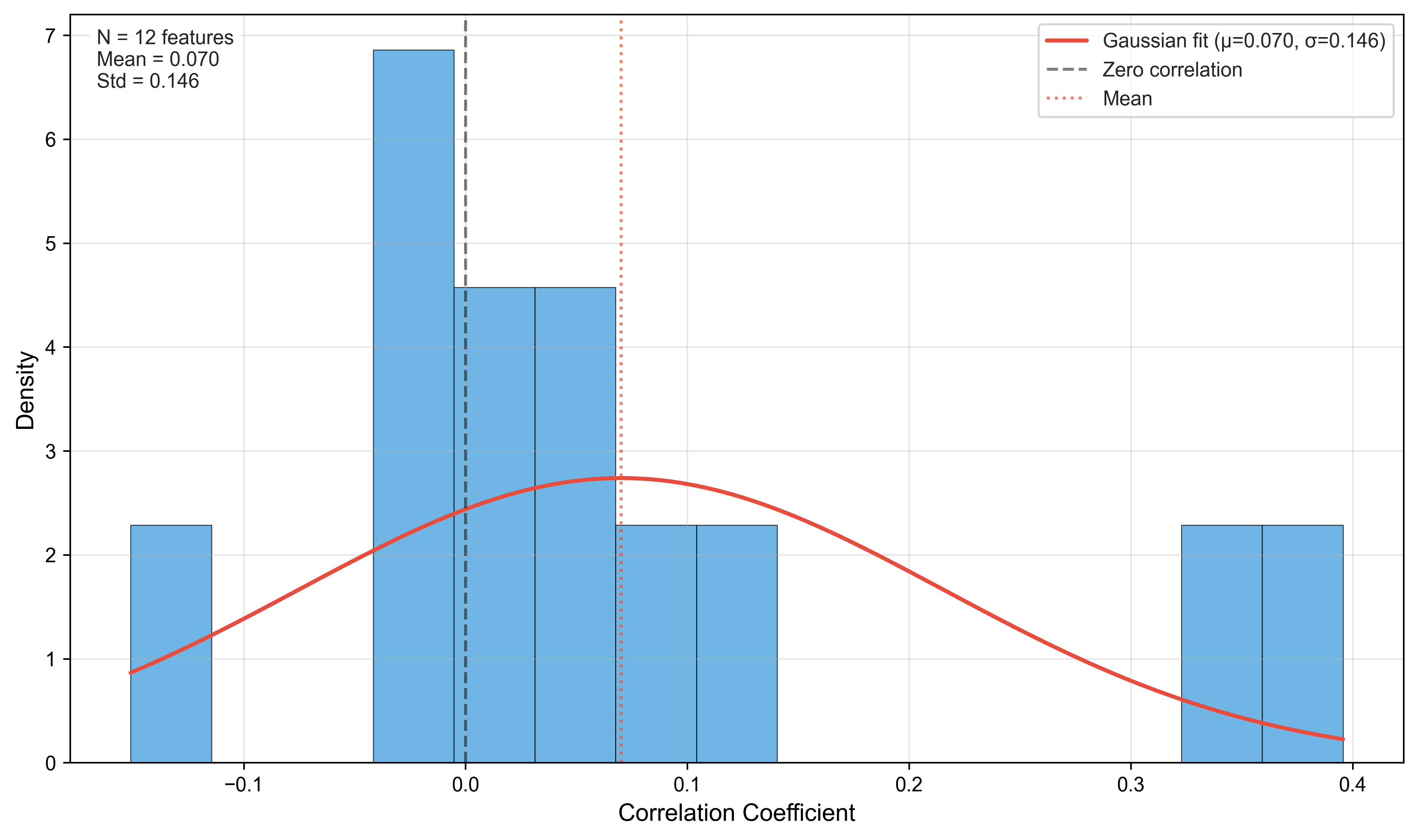

Supplement: S2 Fig — Distribution of Pearson correlation coefficients between 45 episode-level features and IMDb ratings. Sentiment and contestant attributes dominate the upper tail, while task features are near zero. (PNG) [file pone.0331064.s002.png]
