# Supplementary material for: Investigating audience preferences within the hybrid competitive-comedic format of taskmaster UK
Source: PLoS One. 2025 Sep 10;20(9):e0331064. doi: 10.1371/journal.pone.0331064 (PMC12422456; doi:10.1371/journal.pone.0331064)
Supplement: S3 Fig — One-page summaries of all 18 Taskmaster UK series. Each page includes two panels: cumulative contestant score progression (top) and per-task rank evolution (bottom). These visualizations support archetype consistency and highlight turning points in series narratives. (PDF) [file pone.0331064.s003.pdf]

S3 Fig: Series-Level Deep Dive Visualizations

Individual series performance trajectories and archetype assignments for all 18 series of Taskmaster UK

Series 1

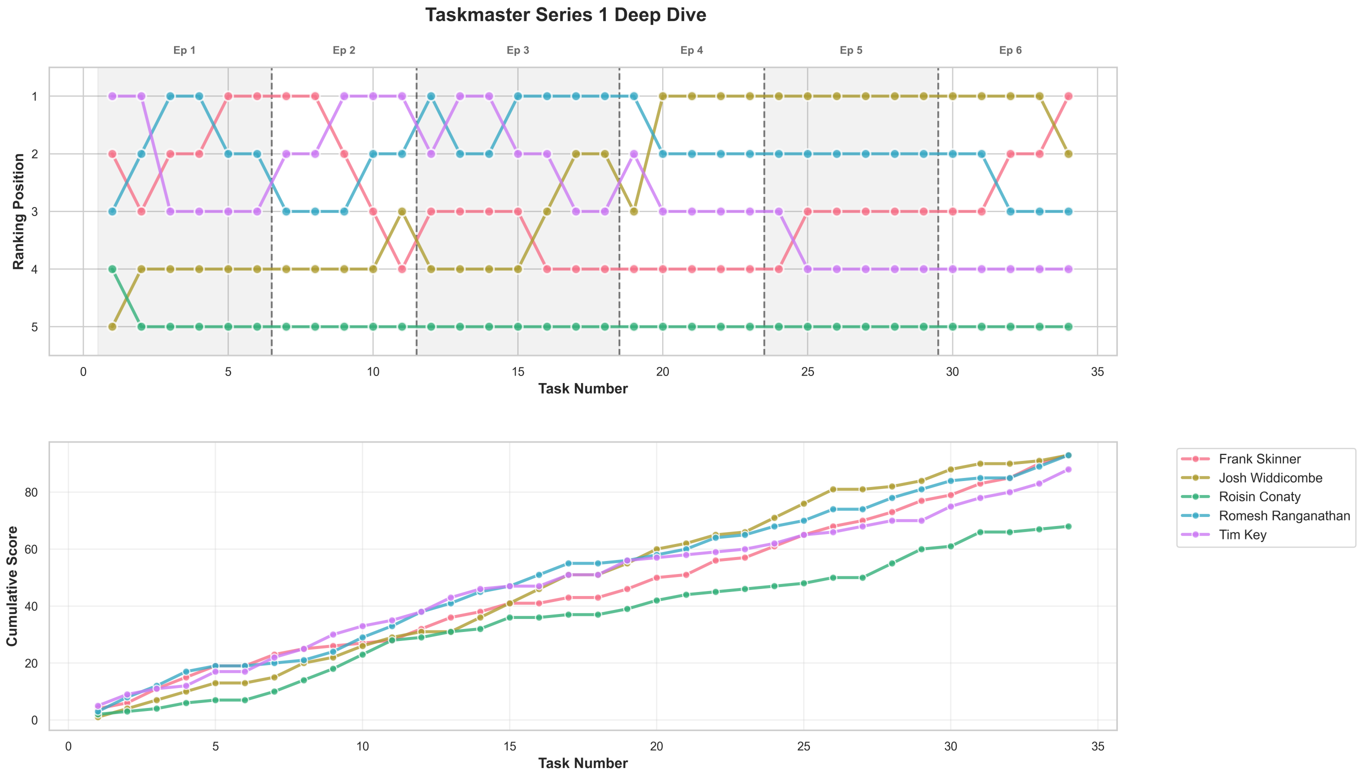

Taskmaster Series 1 Deep Dive
Ep 1
Ep 2
Ep 3
Ep 4
Ep 5
Ep 6
Ranking Position
1
2
3
4
5
0
5
10
15
20
25
30
35
Task Number
Cumulative Score
0
20
40
60
80
Frank Skinner
Josh Widdicombe
Roisin Conaty
Romesh Ranganathan
Tim Key

Series 2

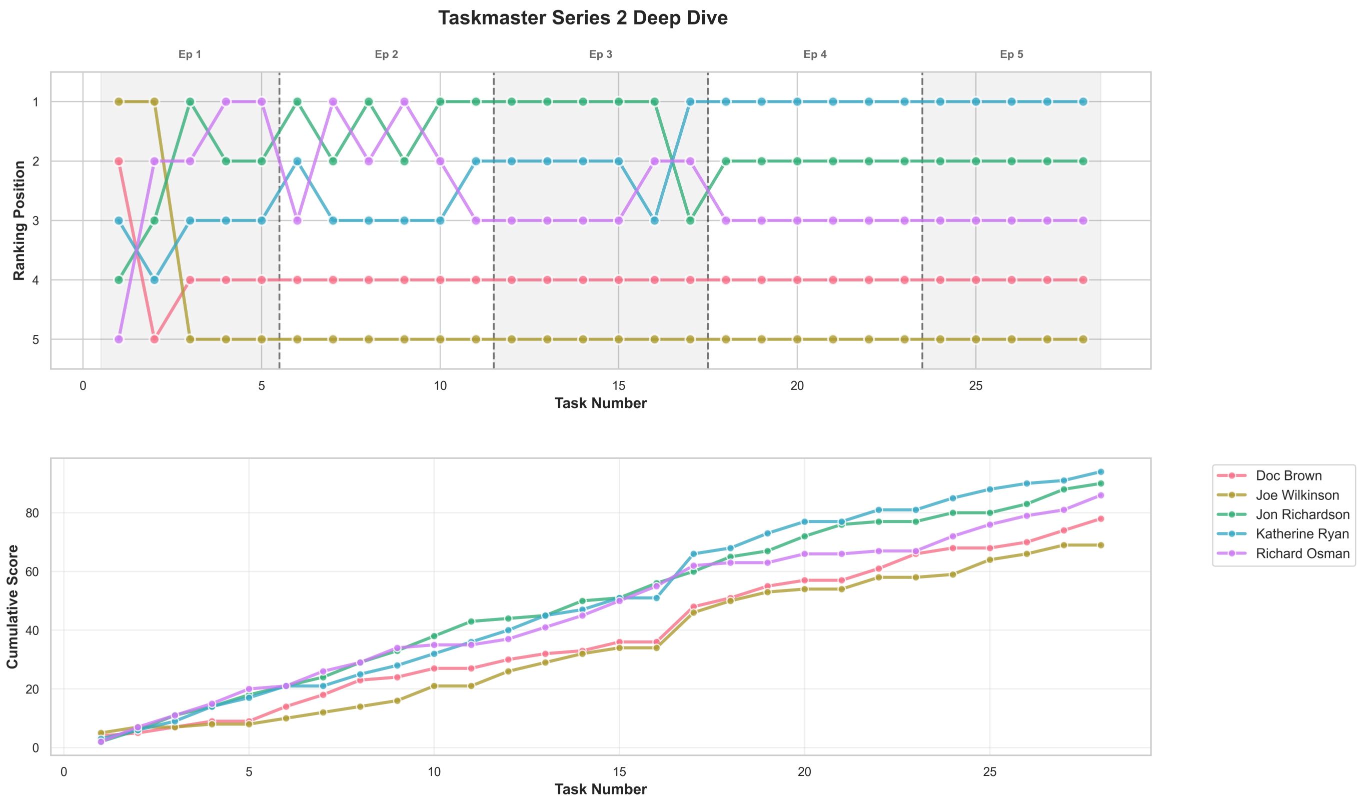

Taskmaster Series 2 Deep Dive
Ep 1
Ep 2
Ep 3
Ep 4
Ep 5
Ranking Position
Task Number
Cumulative Score
Task Number
Doc Brown
Joe Wilkinson
Jon Richardson
Katherine Ryan
Richard Osman

Series 3

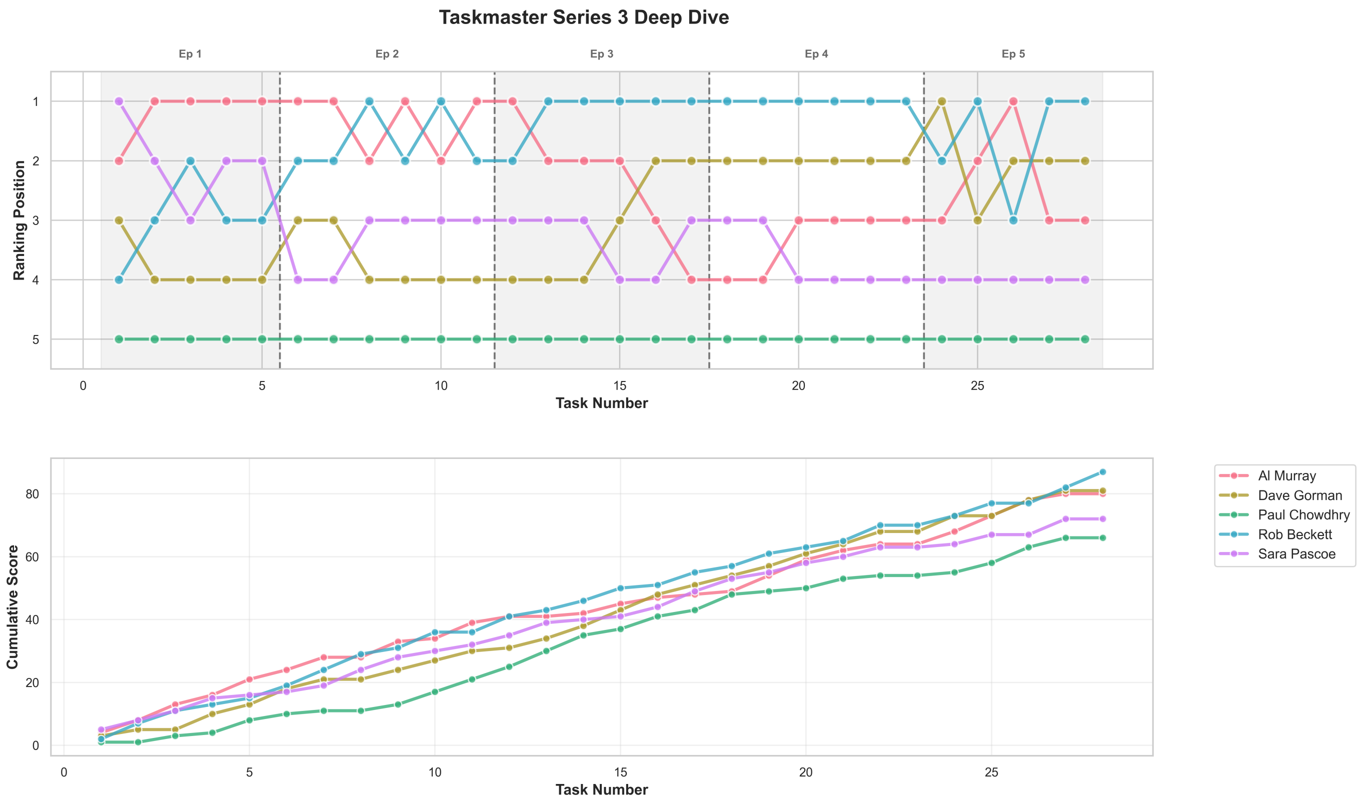
Taskmaster Series 3 Deep Dive
Ep 1
Ep 2
Ep 3
Ep 4
Ep 5
Ranking Position
Task Number
Cumulative Score
Task Number
Al Murray
Dave Gorman
Paul Chowdhry
Rob Beckett
Sara Pascoe

Series 4

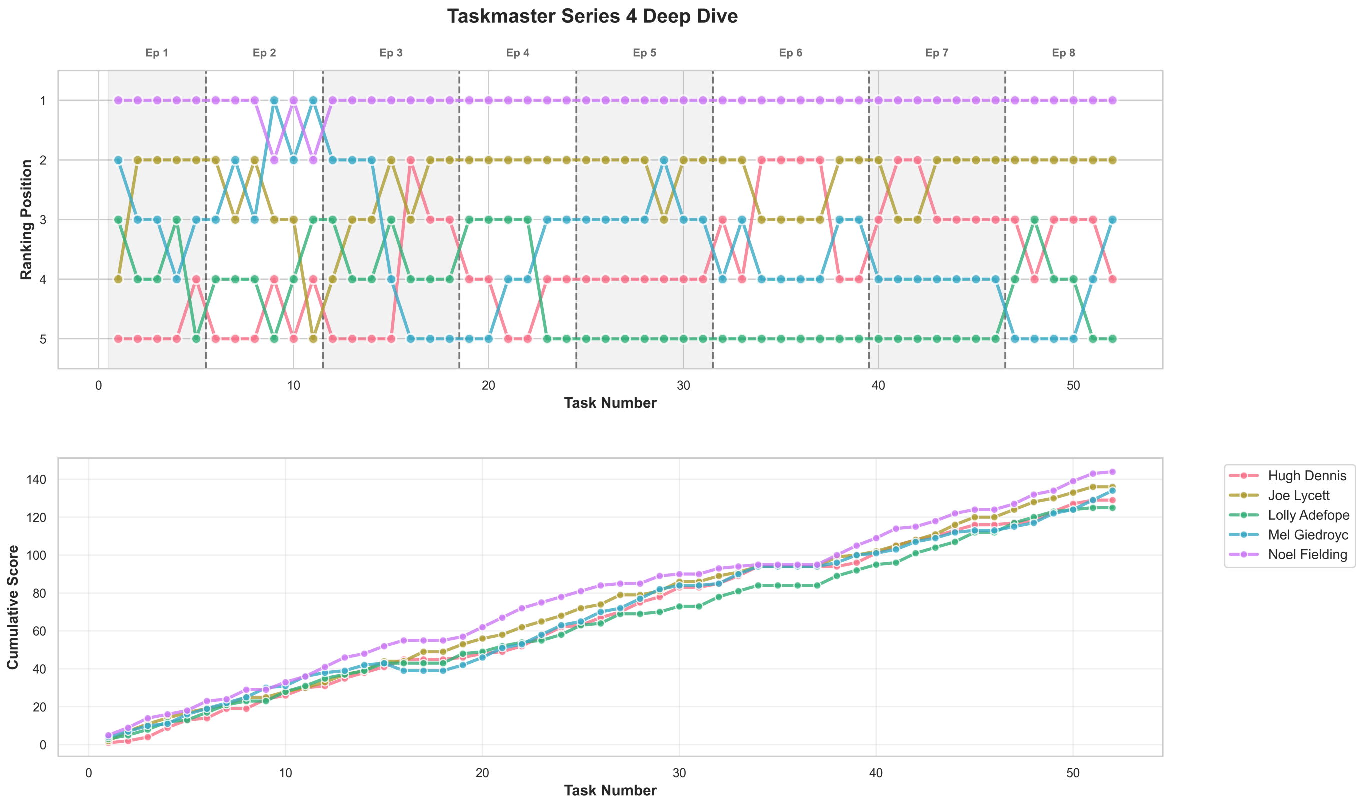
Taskmaster Series 4 Deep Dive
Ep 1
Ep 2
Ep 3
Ep 4
Ep 5
Ep 6
Ep 7
Ep 8
Ranking Position
Task Number
Cumulative Score
Task Number
Hugh Dennis
Joe Lycett
Lolly Adefope
Mel Giedroyc
Noel Fielding

Series 5

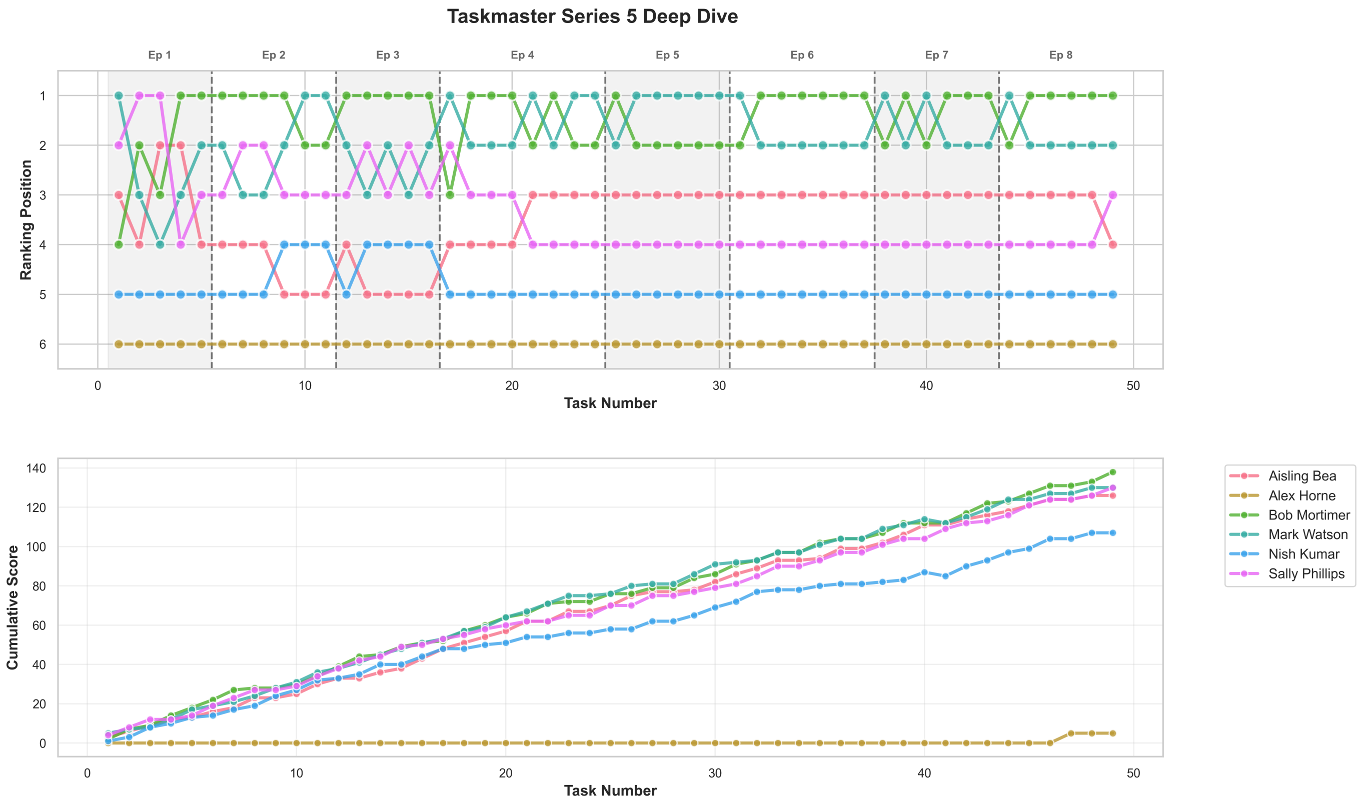
Taskmaster Series 5 Deep Dive
Ep 1
Ep 2
Ep 3
Ep 4
Ep 5
Ep 6
Ep 7
Ep 8
Ranking Position
Task Number
Cumulative Score
Task Number
Aisling Bea
Alex Horne
Bob Mortimer
Mark Watson
Nish Kumar
Sally Phillips

Series 6

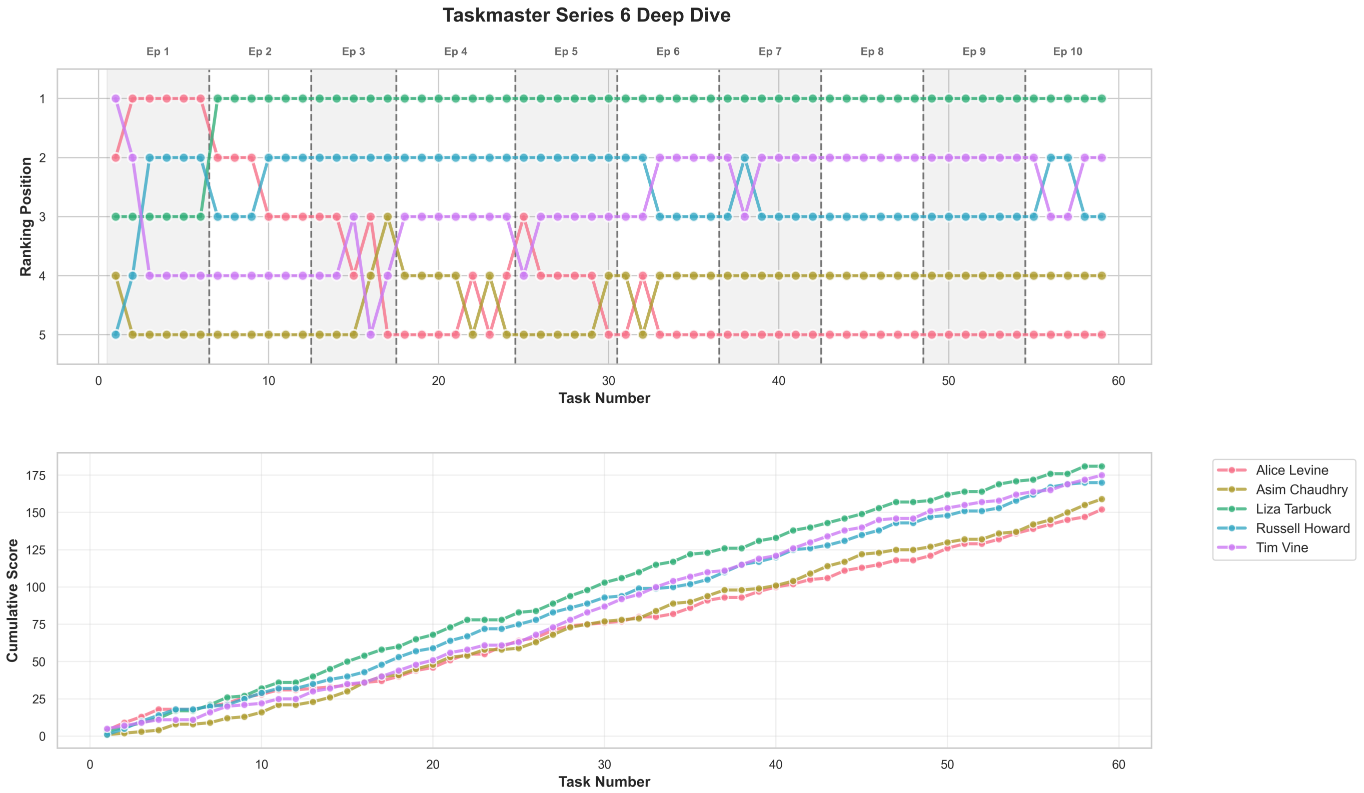

Taskmaster Series 6 Deep Dive
Ep 1
Ep 2
Ep 3
Ep 4
Ep 5
Ep 6
Ep 7
Ep 8
Ep 9
Ep 10
Ranking Position
Task Number
Cumulative Score
Task Number
Alice Levine
Asim Chaudhry
Liza Tarbuck
Russell Howard
Tim Vine

Series 7

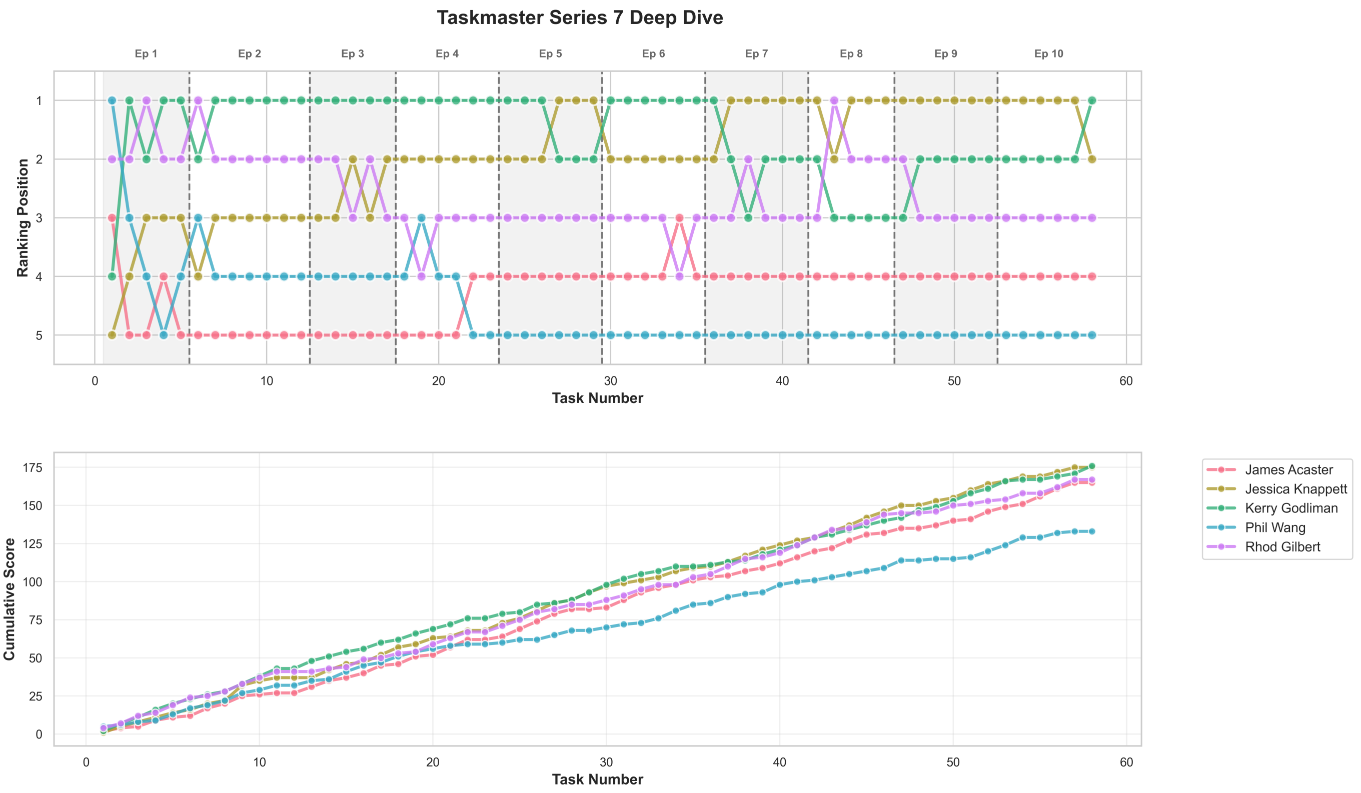
Taskmaster Series 7 Deep Dive
Ep 1
Ep 2
Ep 3
Ep 4
Ep 5
Ep 6
Ep 7
Ep 8
Ep 9
Ep 10
Ranking Position
Task Number
Cumulative Score
Task Number
James Acaster
Jessica Knappett
Kerry Godliman
Phil Wang
Rhod Gilbert

Series 8

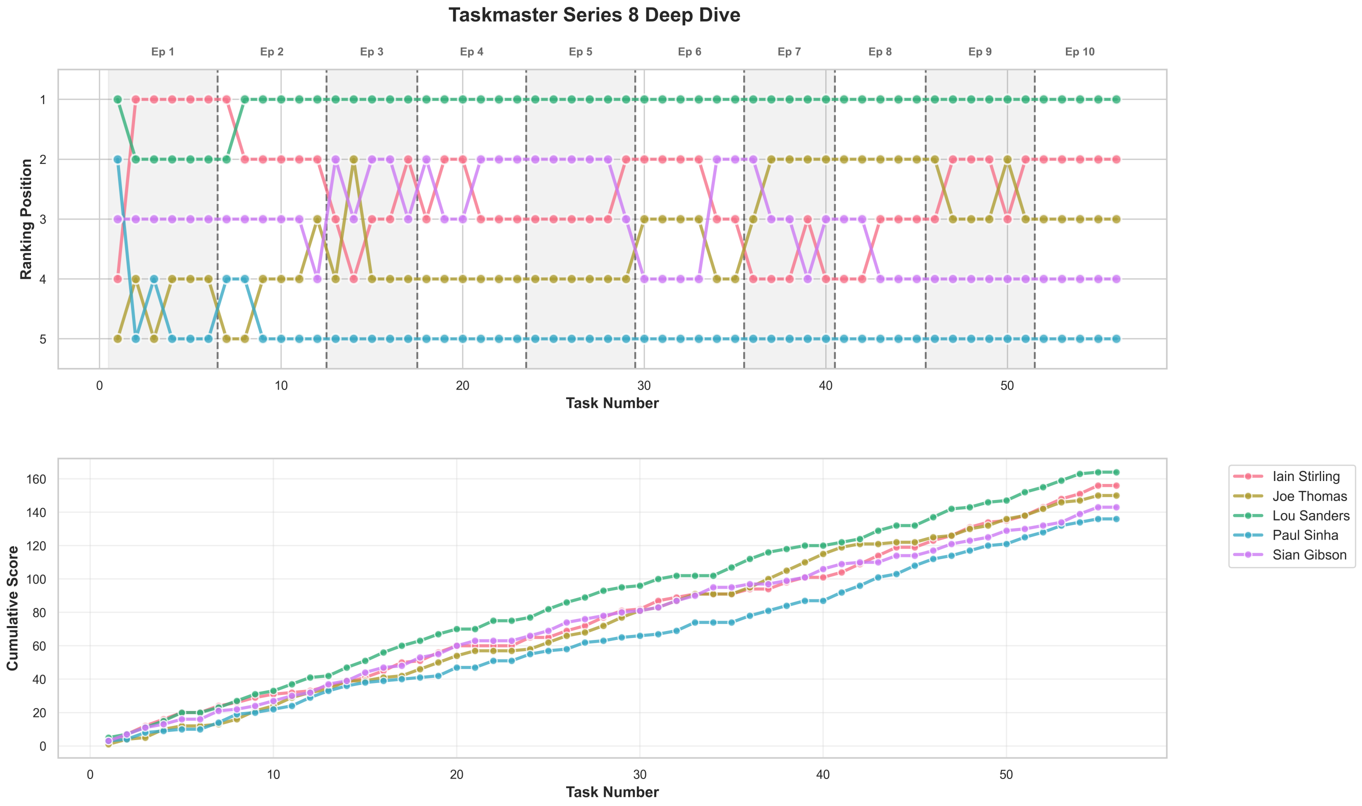
Taskmaster Series 8 Deep Dive
Ep 1
Ep 2
Ep 3
Ep 4
Ep 5
Ep 6
Ep 7
Ep 8
Ep 9
Ep 10
Ranking Position
Task Number
Cumulative Score
Task Number
Iain Stirling
Joe Thomas
Lou Sanders
Paul Sinha
Sian Gibson

Series 9

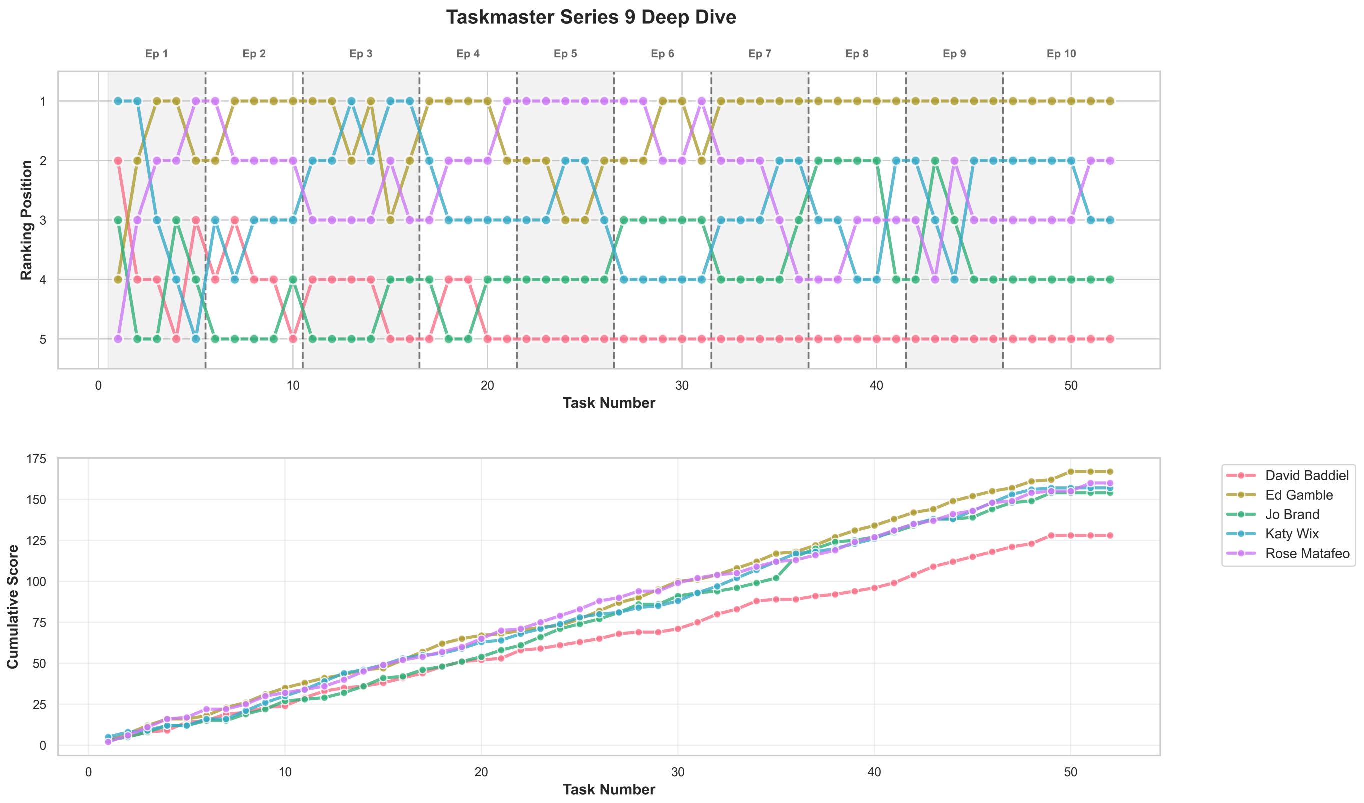

Taskmaster Series 9 Deep Dive
Ep 1
Ep 2
Ep 3
Ep 4
Ep 5
Ep 6
Ep 7
Ep 8
Ep 9
Ep 10
Ranking Position
Task Number
Cumulative Score
Task Number
David Baddiel
Ed Gamble
Jo Brand
Katy Wix
Rose Matafeo

Series 10

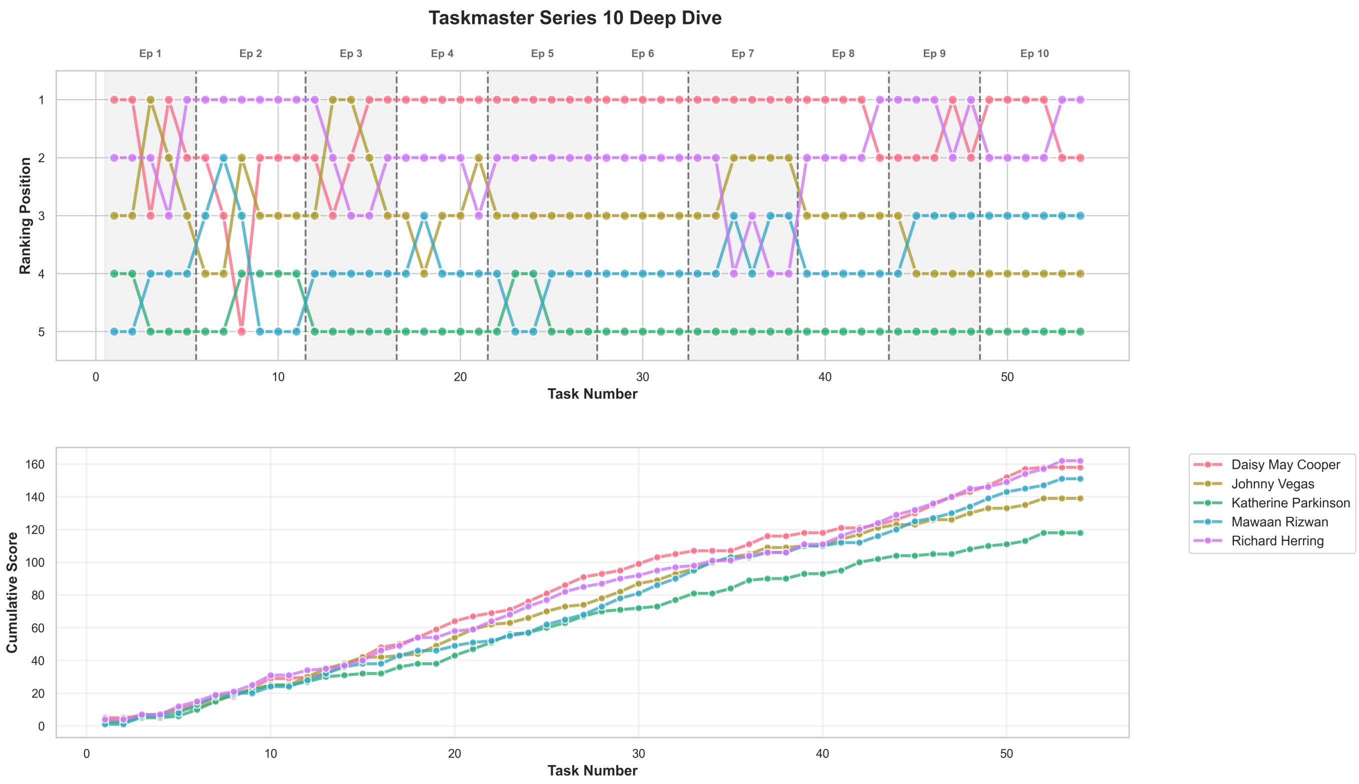
Taskmaster Series 10 Deep Dive
Ep 1
Ep 2
Ep 3
Ep 4
Ep 5
Ep 6
Ep 7
Ep 8
Ep 9
Ep 10
Ranking Position
Task Number
Cumulative Score
Task Number
Daisy May Cooper
Johnny Vegas
Katherine Parkinson
Mawaan Rizwan
Richard Herring

Series 11

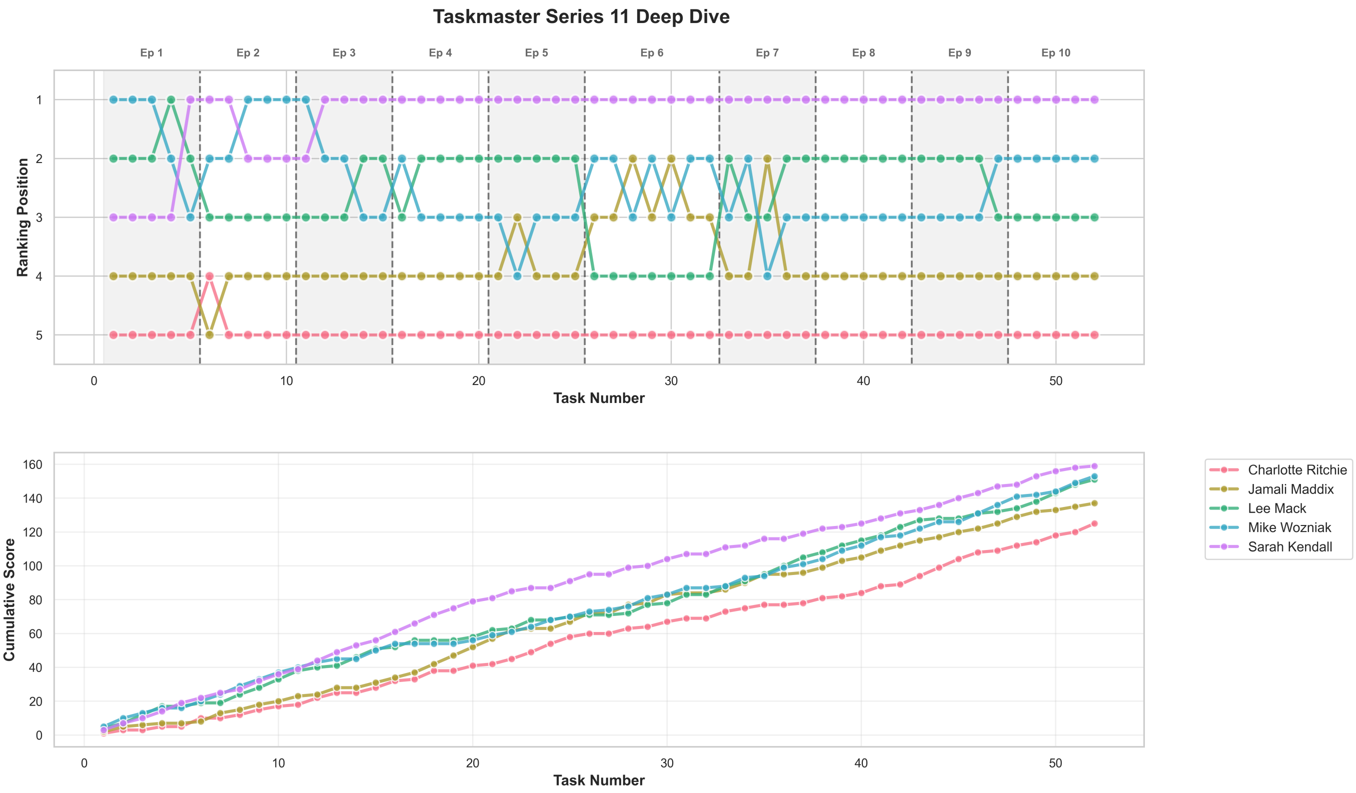
Taskmaster Series 11 Deep Dive
Ep 1
Ep 2
Ep 3
Ep 4
Ep 5
Ep 6
Ep 7
Ep 8
Ep 9
Ep 10
Ranking Position
Task Number
Cumulative Score
Task Number
Charlotte Ritchie
Jamali Maddix
Lee Mack
Mike Wozniak
Sarah Kendall

Series 12

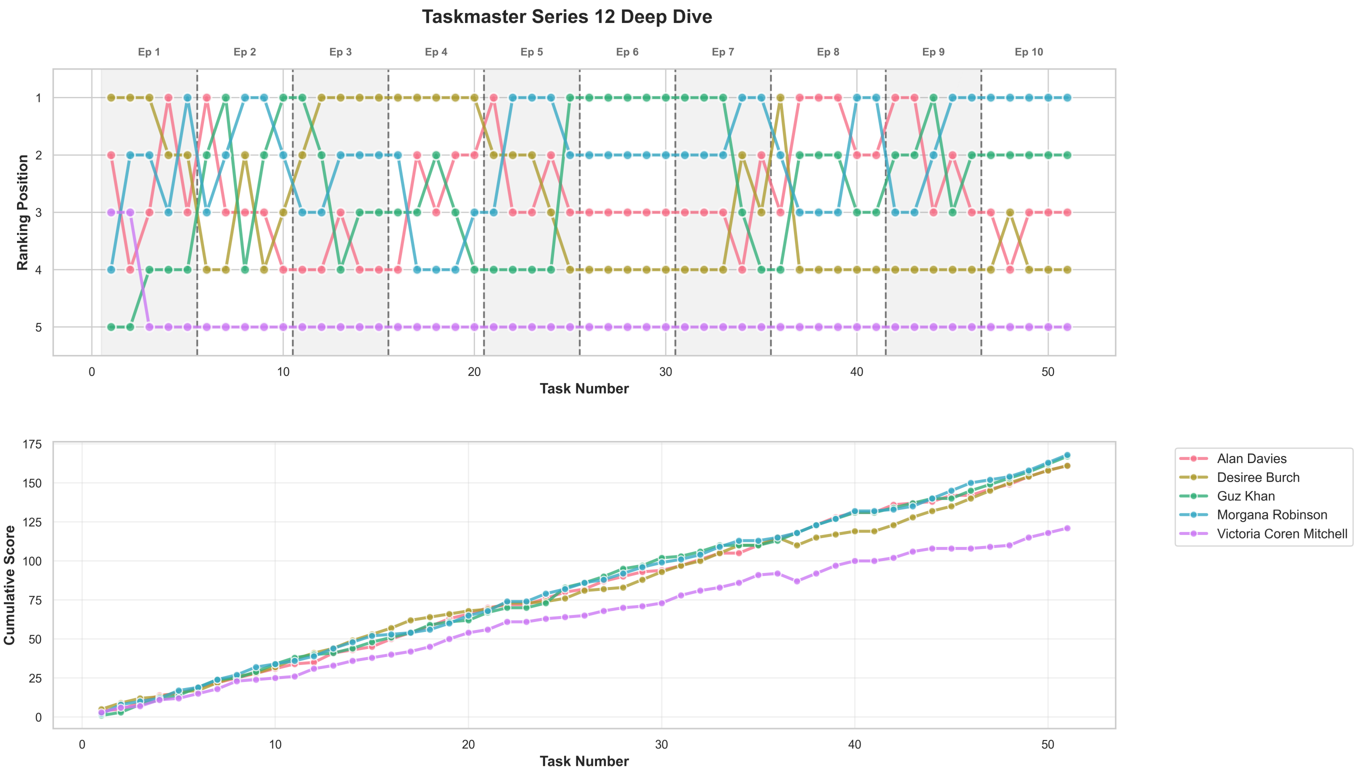
Taskmaster Series 12 Deep Dive
Ep 1
Ep 2
Ep 3
Ep 4
Ep 5
Ep 6
Ep 7
Ep 8
Ep 9
Ep 10
Ranking Position
Task Number
Cumulative Score
Task Number
Alan Davies
Desiree Burch
Guz Khan
Morgana Robinson
Victoria Coren Mitchell

Series 13

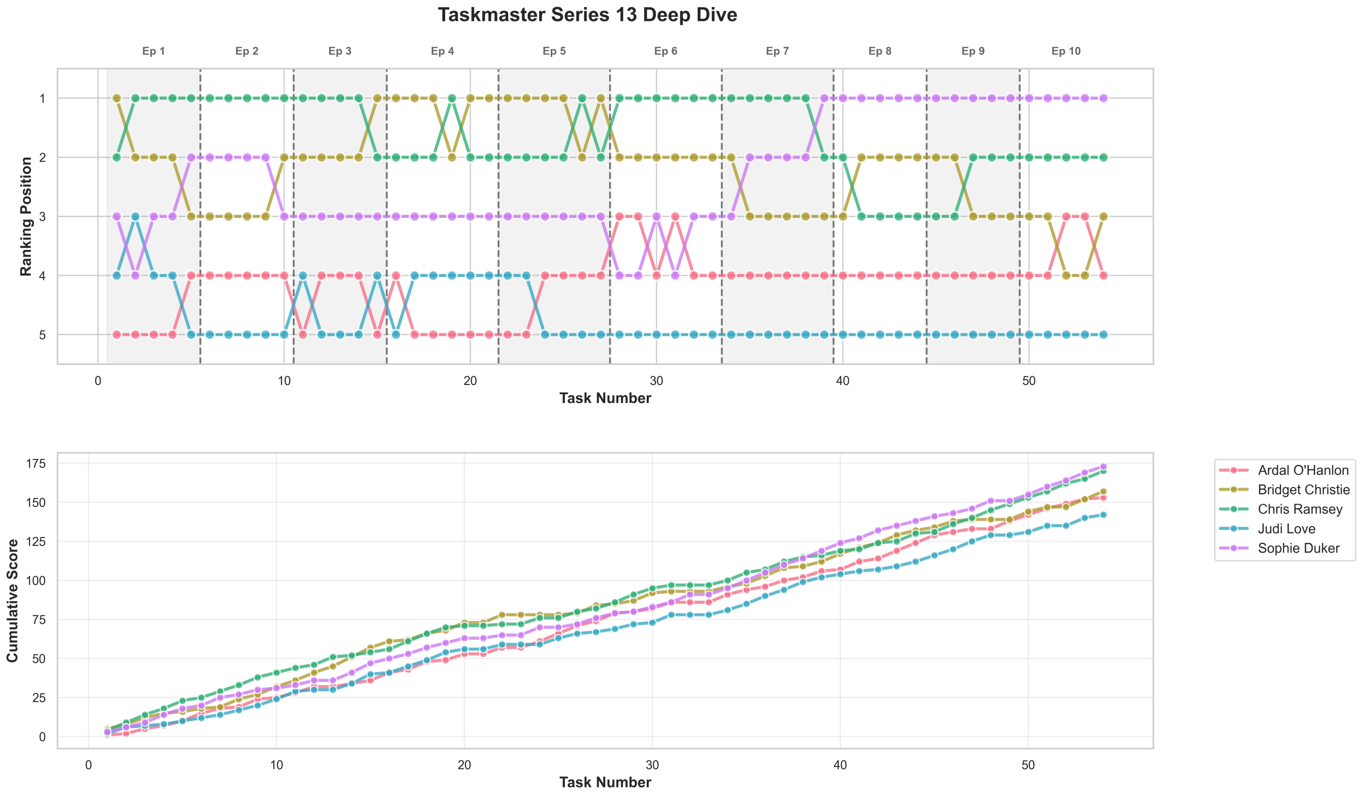

Taskmaster Series 13 Deep Dive
Ep 1
Ep 2
Ep 3
Ep 4
Ep 5
Ep 6
Ep 7
Ep 8
Ep 9
Ep 10
Ranking Position
Task Number
Cumulative Score
Task Number
Ardal O'Hanlon
Bridget Christie
Chris Ramsey
Judi Love
Sophie Duker

Series 14

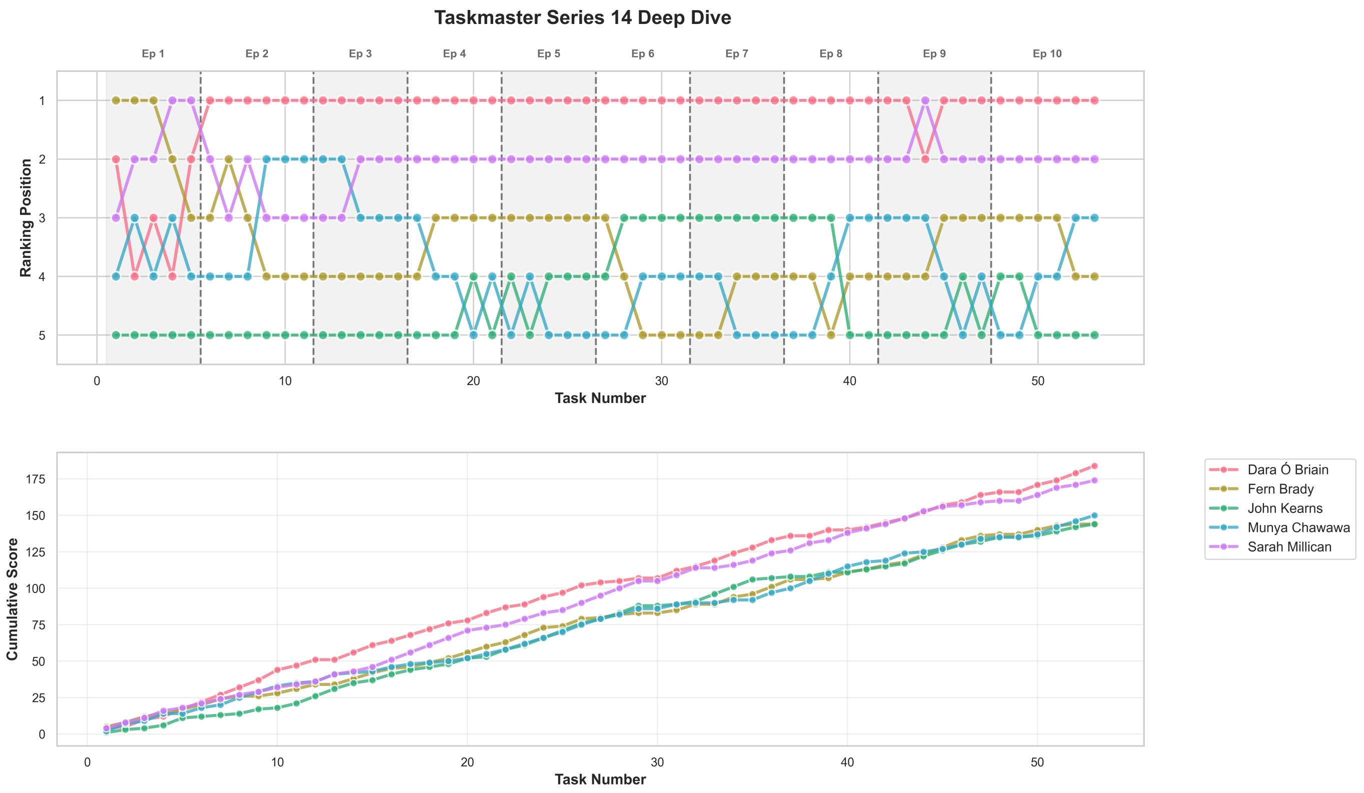

Taskmaster Series 14 Deep Dive
Ep 1
Ep 2
Ep 3
Ep 4
Ep 5
Ep 6
Ep 7
Ep 8
Ep 9
Ep 10
Ranking Position
Task Number
Cumulative Score
Task Number
Dara Ó Briain
Fern Brady
John Kearns
Munya Chawawa
Sarah Millican

Series 15

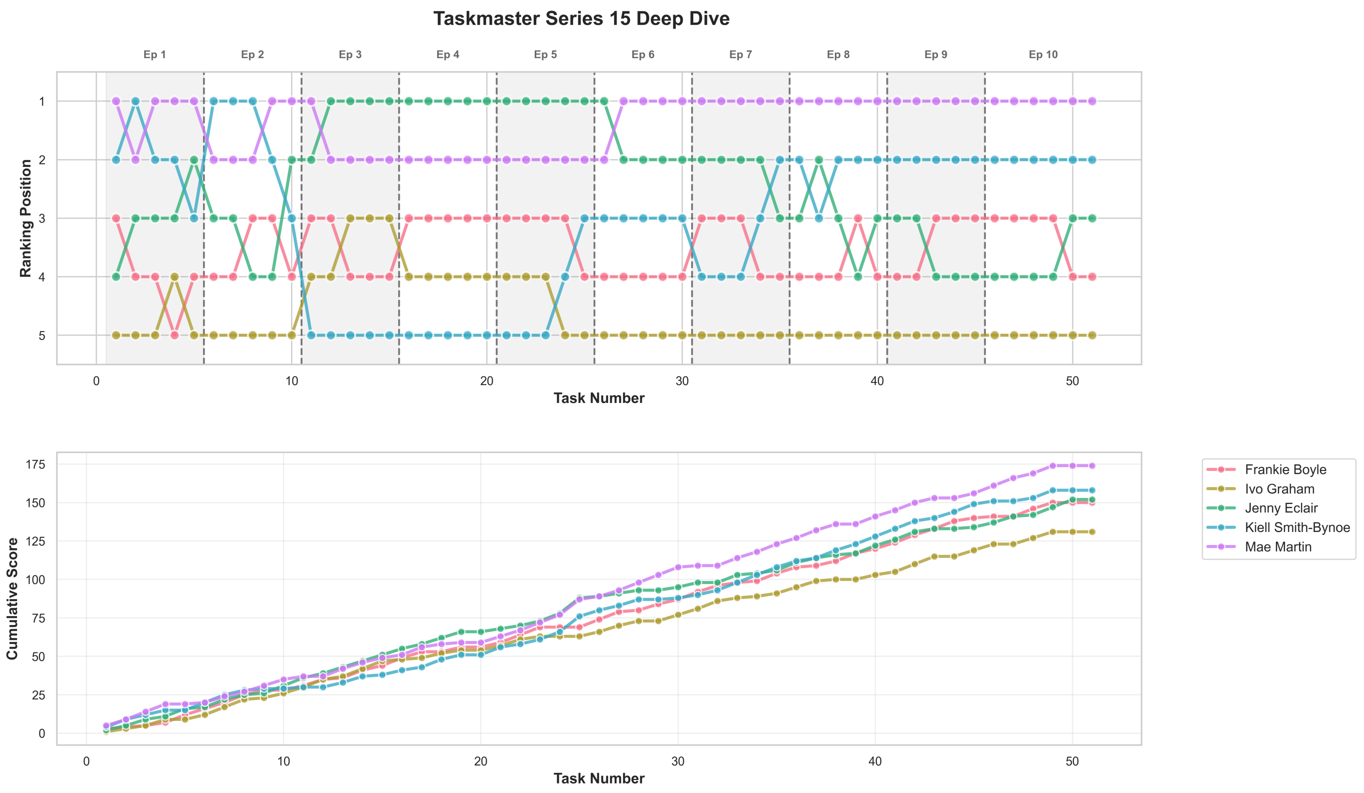

Taskmaster Series 15 Deep Dive
Ep 1
Ep 2
Ep 3
Ep 4
Ep 5
Ep 6
Ep 7
Ep 8
Ep 9
Ep 10
Ranking Position
Task Number
Cumulative Score
Task Number
Frankie Boyle
Ivo Graham
Jenny Eclair
Kiell Smith-Bynoe
Mae Martin

Series 16

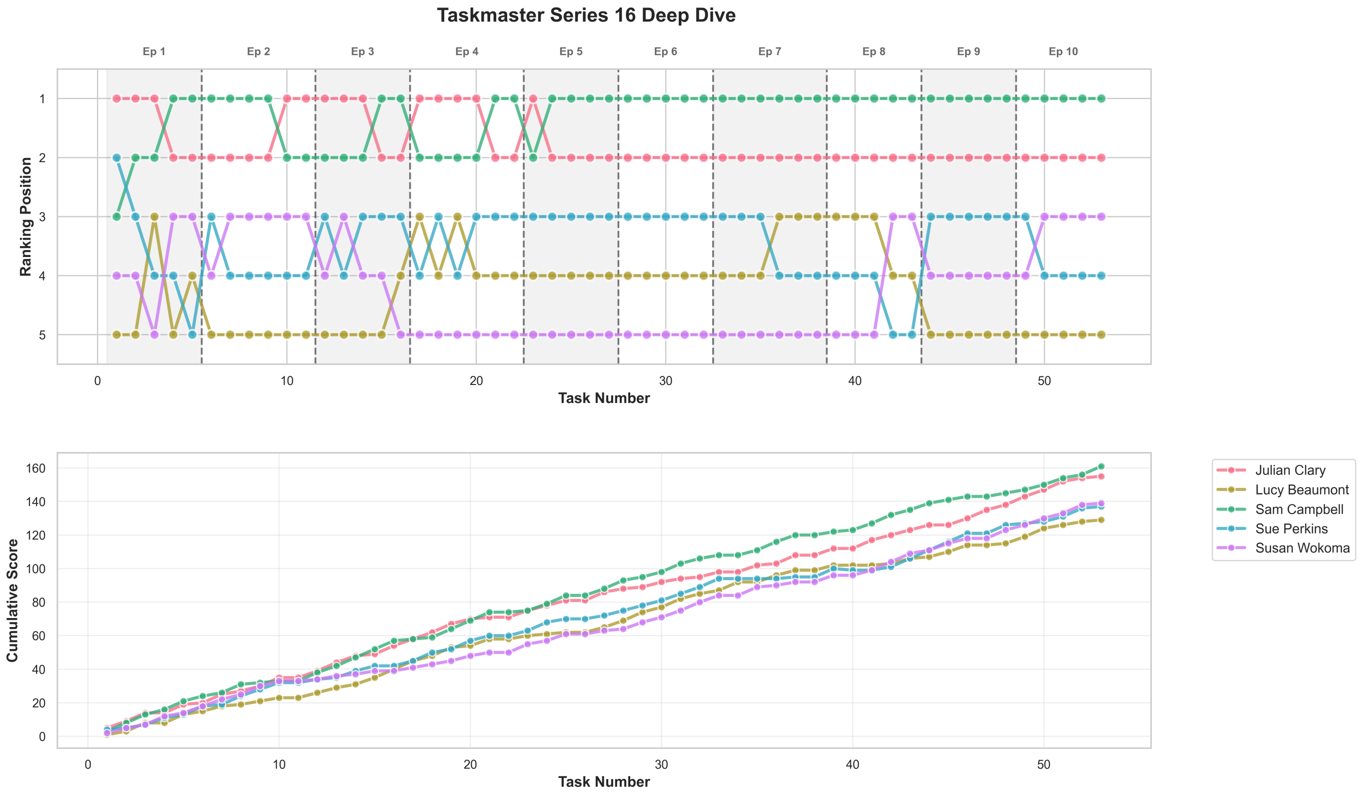
Taskmaster Series 16 Deep Dive
Ep 1
Ep 2
Ep 3
Ep 4
Ep 5
Ep 6
Ep 7
Ep 8
Ep 9
Ep 10
Ranking Position
Task Number
Cumulative Score
Task Number
Julian Clary
Lucy Beaumont
Sam Campbell
Sue Perkins
Susan Wokoma

Series 17

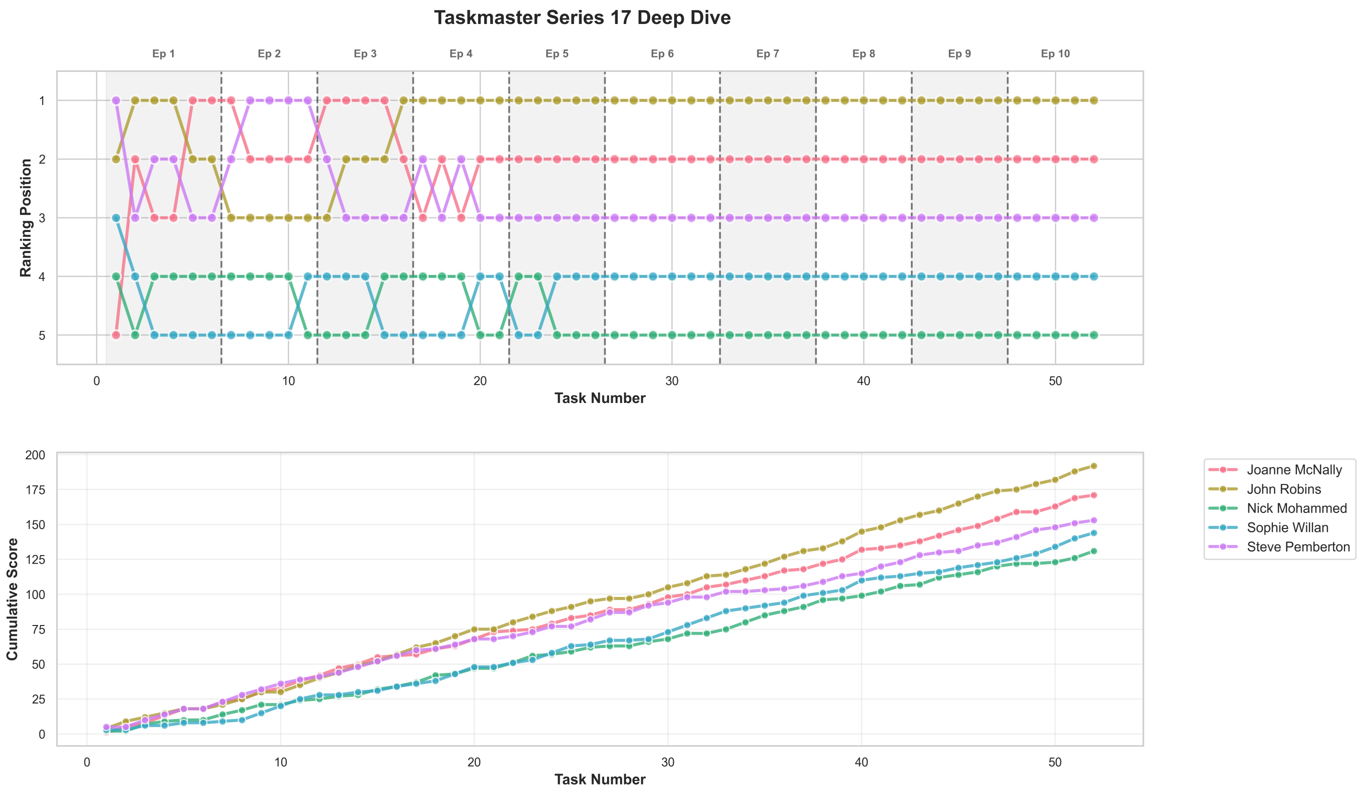
Taskmaster Series 17 Deep Dive
Ep 1
Ep 2
Ep 3
Ep 4
Ep 5
Ep 6
Ep 7
Ep 8
Ep 9
Ep 10
Ranking Position
Task Number
Cumulative Score
Task Number
Joanne McNally
John Robins
Nick Mohammed
Sophie Willan
Steve Pemberton

Series 18

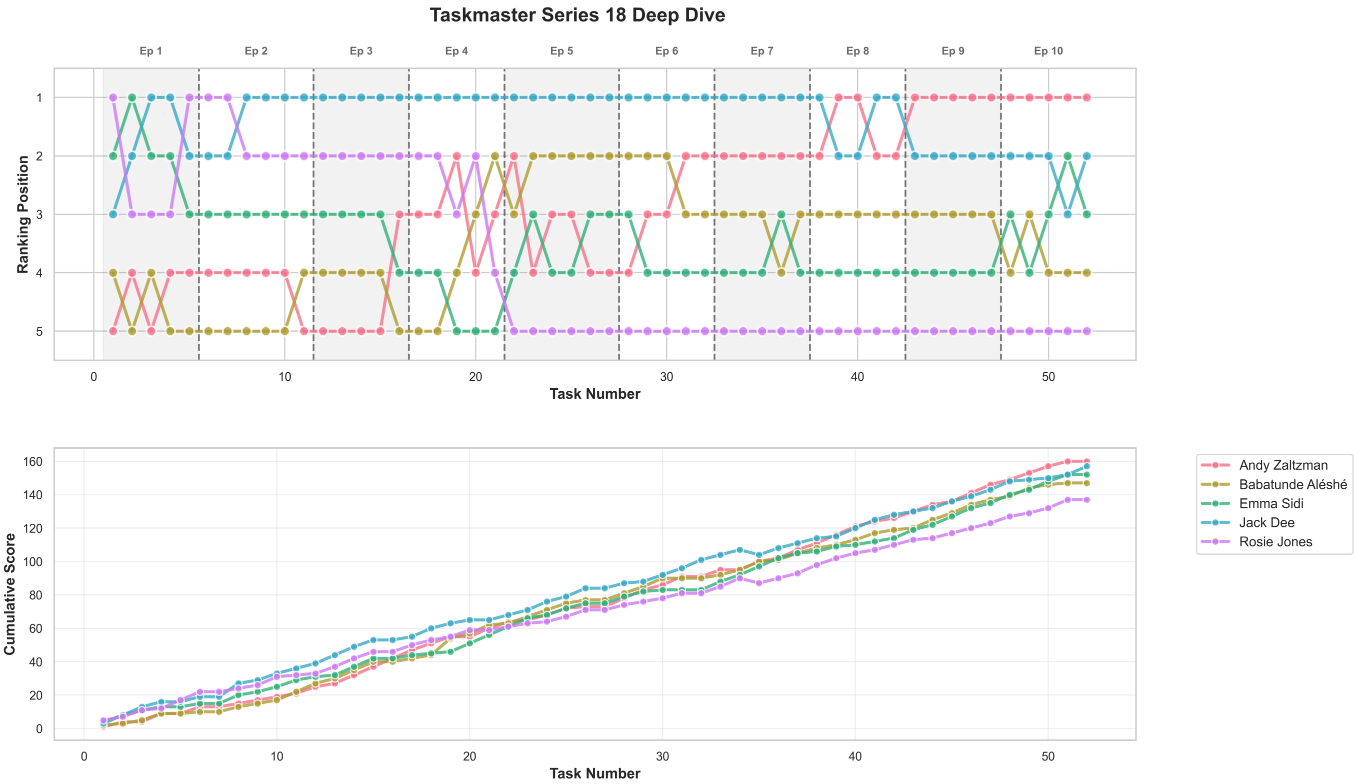
Taskmaster Series 18 Deep Dive
Ep 1
Ep 2
Ep 3
Ep 4
Ep 5
Ep 6
Ep 7
Ep 8
Ep 9
Ep 10
Ranking Position
Task Number
Cumulative Score
Task Number
Andy Zaltzman
Babatunde Aléshé
Emma Sidi
Jack Dee
Rosie Jones
